# Extracellular traps derived from macrophages, mast cells, eosinophils and neutrophils are generated in a time‐dependent manner during atherothrombosis

**DOI:** 10.1002/path.5212

**Published:** 2019-01-25

**Authors:** Kartika R Pertiwi, Onno J de Boer, Claire Mackaaij, Dara R Pabittei, Robbert J de Winter, Xiaofei Li, Allard C van der Wal

**Affiliations:** ^1^ Department of Pathology, Amsterdam UMC University of Amsterdam Amsterdam The Netherlands; ^2^ Department of Biology Education, Faculty of Mathematics and Natural Science Yogyakarta State University Yogyakarta Indonesia; ^3^ Amsterdam Heart Centre, Amsterdam UMC Amsterdam The Netherlands; ^4^ Department of Pathology Maastricht UMC Maastricht The Netherlands

**Keywords:** extracellular traps, neutrophil, macrophage, mast cell, eosinophil, atherosclerosis, coronary thrombosis, myocardial infarction

## Abstract

Extracellular traps generated by neutrophils contribute to thrombus progression in coronary atherosclerotic plaques. It is not known whether other inflammatory cell types in coronary atherosclerotic plaque or thrombus also release extracellular traps. We investigated their formation by macrophages, mast cells, and eosinophils in human coronary atherosclerosis, and in relation to the age of thrombus of myocardial infarction patients. Coronary arteries with thrombosed or intact plaques were retrieved from patients who died from myocardial infarction. In addition, thrombectomy specimens from patients with myocardial infarction were classified histologically as fresh, lytic or organised. Neutrophil and macrophage extracellular traps were identified using sequential triple immunostaining of CD68, myeloperoxidase, and citrullinated histone H3. Eosinophil and mast cell extracellular traps were visualised using double immunostaining for eosinophil major basic protein or tryptase, respectively, and citrullinated histone H3. Single‐ and double‐stained immunopositive cells in the plaque, adjacent adventitia, and thrombus were counted. All types of leucocyte‐derived extracellular traps were present in all thrombosed plaques, and in all types of the *in vivo‐*derived thrombi, but only to a much lower extent in intact plaques. Neutrophil traps, followed by macrophage traps, were the most prominent types in the autopsy series of atherothrombotic plaques, including the adventitia adjacent to thrombosed plaques. In contrast, macrophage traps were more numerous than neutrophil traps in intact plaques (lipid cores) and organised thrombi. Mast cell and eosinophil extracellular traps were also present, but sparse in all instances. In conclusion, not only neutrophils but also macrophages, eosinophils, and mast cells are sources of etosis involved in evolving coronary thrombosis. Neutrophil traps dominate numerically in early thrombosis and macrophage traps in late (organising) thrombosis, implying that together they span all the stages of thrombus progression and maturation. © 2018 The Authors. *The Journal of Pathology* published by John Wiley & Sons Ltd on behalf of Pathological Society of Great Britain and Ireland.

## Introduction

Extracellular traps (ETs) are thread‐like structures of decondensed DNA decorated with proteins from cytoplasmic granules 1, 2. Neutrophil extracellular traps (NETs) were the first that were identified, and it was found that these structures are involved in the elimination of pathogens 3. The process of ET formation is called ‘etosis’, which is a specific type of cell death 4. Nowadays, there is increasing interest in the role of NETs also in autoimmune and cardiovascular diseases 5. Several studies have documented their presence in human atherosclerotic plaques and thrombosis 6, 7, 8, 9, 10. NETs are believed to promote endothelial dysfunction 1; to stimulate thrombus formation on disrupted plaque, mainly by providing scaffolds for fibrin deposition 11; and to stabilise clot formation through the activation of coagulation cascade and thrombin generation, via their bearing of tissue factor (TF) 9, 12.

Recent research has shown that ETs can also be generated by cells other than neutrophils 13, such as macrophages 14, 15, 16, mast cells 17, 18, and eosinophils 19, 20; these are termed macrophage extracellular traps (METs), mast cell extracellular traps (MCETs), and eosinophil extracellular traps (EETs), respectively. METs, MCETs, and EETs have been identified in infectious 15, 18, 21, 22 and autoimmune diseases 17, 19. Given the abundant presence of macrophages 23, 24, 25, and to a lesser extent also mast cells 26, 27 and eosinophils in atherosclerotic plaques 28, 29, we hypothesised that these structures may contribute to atherothrombosis, and in particular to the process of thrombosis resulting from plaque rupture or erosion. Therefore, we investigated the presence and relative extent of ETs released by macrophages (METs), mast cells (MCETs), and eosinophils (EETs) in intact and thrombosed (eroded and ruptured) coronary atherosclerotic plaques of autopsied patients who had died of acute myocardial infarction (MI). In addition, we evaluated whether the occurrence of different types of etosis also relates to the age of a coronary thrombus in MI patients. For this purpose, we used coronary thrombectomy materials, in which the age of the thrombus may vary substantially from fresh (representing recent‐onset thrombus) to cell‐rich organised masses (representing thrombus that is weeks old) 30. For quantitative comparison of the extent of different ET types within the plaques and the thrombi, we also included neutrophils and NETs in this study, as previously reported 6, 31.

## Materials and methods

### Specimen selection and classification

#### Autopsy materials

Paraffin blocks containing coronary plaques obtained at autopsy from patients with acute MI were derived from the pathology archives of the Academic Medical Center, Amsterdam. For the purpose of this study, six plaques with intact endothelial surface without thrombus and 12 thrombosed plaques were selected, of which the latter had either a fibrous cap rupture (*n* = 6) or plaque erosion underlying the thrombus (*n* = 6).

#### Thrombectomy specimens

Paraffin blocks containing thrombus aspiration materials derived from MI patients were retrieved from the pathology archives of the Academic Medical Center, Amsterdam. The retrieved thrombus blocks were cut into 5‐μm‐thick sections and histomorphologically graded on H&E‐stained sections according to the age of thrombus into three categories – fresh, lytic, and organised – as previously described 30, 32, 33. Fresh thrombus (up to 1 day) was composed of intact platelets, erythrocytes, and/or granulocytes; lytic thrombus (1–5 days) was identified by the presence of colliquation necrosis and karyorrhexis of granulocytes; and organised thrombus (> 5 days) was marked by the appearance of (myo)fibroblasts and extracellular matrix deposits. Thrombus materials with a mixed composition of different ages were separately graded. From the total file of archived specimens, we randomly selected 48 specimens, resulting in 24 fresh, 26 lytic, and 18 organised thrombi for further immunohistochemistry in this study.

Criteria for the proper secondary use of human tissue in The Netherlands were met and accordingly the AMC Medical Ethical Board grants a waiver for the use of ‘left‐over materials’ that are used anonymously.

### Immunohistochemistry

Immunostaining was performed using the following antibodies: anti‐MPO for neutrophils (myeloperoxidase, A0398; Dako, Glostrup, Denmark; dilution 1:5000), CD68 for macrophages (clone PG‐M1, M0876; Dako; dilution 1:200), tryptase for mast cells (clone AA1, M7052; Dako; dilution 1:5000), EMBP (eosinophil major basic protein) for eosinophils (clone BMK‐13, MON6008‐1; MonoSan, Funakoshi, Tokyo, Japan; dilution 1:50); and CitH3 (citrullinated histone‐3) for ETs (ab5103; Abcam, Cambridge, UK; dilution 1:4000). NETs and METs were identified using sequential triple staining of anti‐CD68, MPO, and CitH3 (see supplementary material, Figure S1), whereas EETs and MCETs were visualised using sequential double staining of anti‐EMBP or tryptase with anti‐CitH3, respectively. Antigen retrieval was performed with heat‐induced antigen retrieval (Lab Vision™ PT Module; Thermo Fisher Scientific, Fremont, CA, USA) using Tris‐EDTA buffer (Thermo Fisher Scientific). For the secondary antibody, polymer horseradish peroxidase (HRP) anti‐rabbit or anti‐mouse (ImmunoLogic, Duiven, The Netherlands) was used and the immune complexes were detected using Vector Nova Red (Vector Laboratories, Burlingame, CA, USA) as chromogen. After each staining round, our sequential immunostaining protocol required the stained sections to be digitised using a slide scanner (Philips IntelliSite UFS; Philips Digital Pathology Solutions, Best, The Netherlands), followed by an elution step to remove the dye and immune complexes using stripping buffer, as previously described 6. Positive and negative controls (omission of the primary antibody) were always included (see supplementary material, Figure S2). Furthermore, control experiments for the staining of ETs consisted of pretreating the sections with DNase I (Thermo Fisher Scientific) as previously described 31.

### Quantification of immunostaining results

Digitised immunostained sections were downloaded from the Philips image management system. Using the downloaded images, we further investigated the areas of interest for quantification. For plaque specimens, the number of immunopositive cells was counted in different topographic locations: plaques (intact plaques), thrombus and adjacent plaque (thrombosed plaques), and adventitia (both plaque types); whereas for coronary thrombi, these were quantified in areas of interest according to the distinct features of each histological thrombus age. The surface areas of the selected regions were measured in mm^2^. The separate downloaded images were then digitally aligned by a non‐linear registration as described previously 6, creating image stacks to calculate the immunopositive cell‐specific (MPO, CD68, tryptase or EMBP) antibodies and their co‐localisation with CitH3. NETs were determined as MPO^+^CD68^−^CitH3^+^ cells, while METs were either CD68^+^MPO^−^CitH3^+^ or CD68^+^MPO^+^CitH3^+^ cells (see supplementary material, Figure S1). In terms of MCETs and EETs, they were defined as tryptase^+^CitH3^+^ cells or EMBP^+^CitH3^+^ cells, respectively. All measurements were expressed as number of cells per mm^2^.

### Statistics

Statistical analysis was conducted using SPSS 24.00 (IBM Corporation, Armonk, NY, USA). According to the normal or non‐normal distribution of the data, we performed either a *t*‐test or a Mann–Whitney test for comparison between thrombosed and intact plaques, and either Kruskal Wallis or ANOVA for different thrombus ages with a *post hoc* test when the results were significant (a value of *p* < 0.05 was considered significant).

## Results

### Extracellular traps in intact and thrombosed atherosclerotic plaques at autopsy

**Figure 1:**
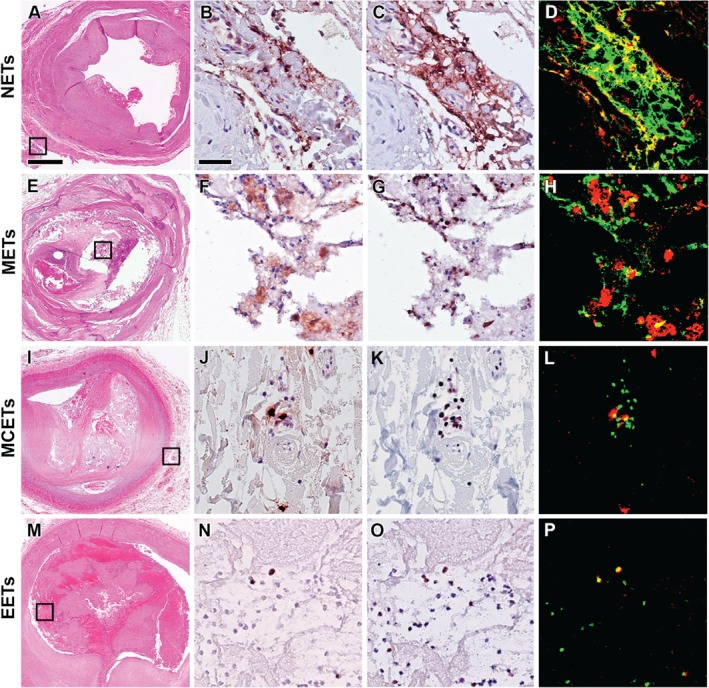
Extracellular traps (ETs) in coronary plaques. Representative examples of neutrophil, macrophage, mast cell, and eosinophil extracellular traps (NETs, METs, MCETs, and EETs) in coronary plaques with thrombotic complications (A–D, I–P: erosions; E–H: ruptures). As previous reports have demonstrated that macrophages can produce MPO, a triple staining strategy was used to differentiate METs from NETs (see the Materials and methods section), but as there were few CD68^+^/MPO^+^ cells, only dual staining with citH3 is shown. Boxed areas in the H&E stains (A, E, I, and M) show the regions of interest for higher magnification of immunostaining with cell‐specific antibodies: anti‐MPO for neutrophils (B), CD68 for macrophages (F), tryptase for mast cells (J), EMBP for eosinophils (N), and with anti‐citH3 antibodies for ETs (C, G, K, and O), showing positive cells in brown/dark red, as well as of false‐colour images to show the co‐localisation of CitH3^+^ (in green) with either neutrophils^+^, macrophages^+^, mast cells^+^ or eosinophils^+^ (in red) as NETs (D), METs (H), MCETs (L) or EETs (P), in yellow, respectively. Scale bar in H&E overview (A): 100 μm and in high‐power detail (B): 25 μm.

**Figure 2:**
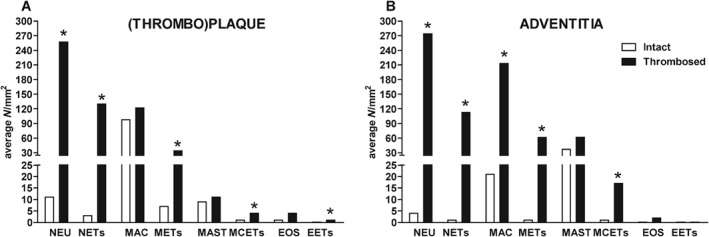
Quantification of neutrophil, macrophage, mast cell, and eosinophil extracellular traps (NETs, METs, MCETs, and EETs) in intact and thrombosed coronary plaques. Number of immunopositive cells per surface area (mm^2^) stained for neutrophils (NEU), macrophages (MAC), mast cells (MAST), and eosinophils (EOS); and number of NETs, METs, MCETs, and EETs per surface area (mm^2^) in (thrombo)plaque (A) and in adventitia (B). A higher number of NETs, METs, MCETs, and EETs were encountered in thrombosed than in intact plaques (*p < 0.05, *n* = 18).

The occurrence of NETs, METs, MCETs, and EETs was noticed in all 12 coronary segments with thrombosed plaques. They were present in different locations: not only in the plaque but also in adjacent thrombus and in surrounding perivascular fat (see Figure 1 for representative examples of NETs, METs, MCETs, and EETs in plaques). METs were the most numerous type of extracellular trap inside the intact (not thrombosed) plaques (Figure 2A), and were located mostly around the lipid core of lesions. MCETs and EETs were only sparsely present in this location (Figure 2A). In the adjacent adventitia and perivascular fat of these intact plaques, not only NETs but also METs and MCETs were low in number, and EETs were almost never encountered (Figure 2B). When compared with these intact atherosclerotic plaques, the average number of NETs, METs, MCETs, and EETs was significantly increased in the plaques with thrombotic complications, either in the (thrombo)plaque or in the adventitia and perivascular fat (*p* < 0.05, Figure 2A,B). In addition, the majority of ETs in thrombosed plaques were neutrophil‐derived, followed by macrophage‐, mast cell‐ and eosinophil‐derived traps.

### Extracellular traps in coronary thrombus aspirates

**Figure 3:**
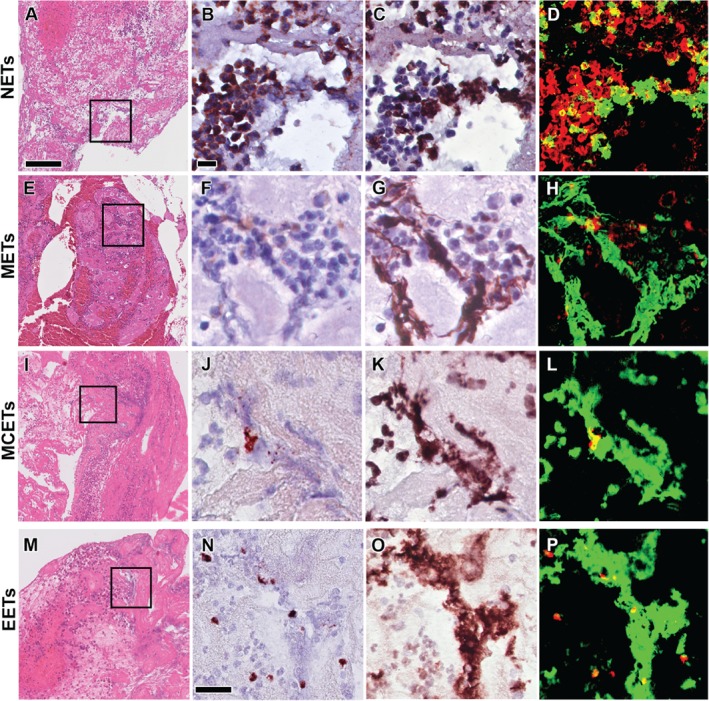
Extracellular traps (ETs) in coronary thrombi. Representative examples of neutrophil, macrophage, mast cell, and eosinophil extracellular traps (NETs, METs, MCETs, and EETs) in coronary thrombi (A–D, I–P: lytic; E–H: fresh). Boxed areas in the H&E stains (A, E, I, and M) show the regions of interest for higher magnification of immunostaining with cell‐specific antibodies: anti‐MPO for neutrophil (B), anti‐CD68 for macrophages (F), anti‐tryptase for mast cells (J), and anti‐EMBP for eosinophils (N), and with anti‐citH3 antibodies for ETs (C, G, K, and O), showing positive cells in brown/dark red, as well as of false‐colour images to show the co‐localisation of CitH3^+^ (in green) with either neutrophils^+^, macrophages^+^, eosinophils^+^ or mast cells^+^ (in red) as NETs (D), METs (H), MCETs (L) or EETs (P), in yellow, respectively. Scale bar in H&E overview (A): 100 μm and in high‐power detail (B, N): 25 μm.

**Figure 4:**
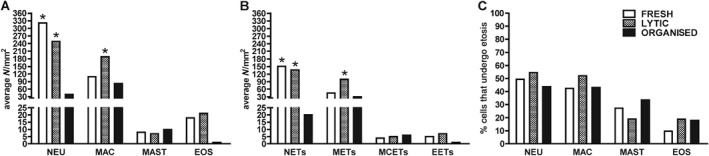
Quantification of neutrophil, macrophage, mast cell, and eosinophil extracellular traps (NETs, METs, MCETs, and EETs) in relation to the age of coronary thrombi of myocardial infarction patients. (A) Number of immunopositive cells per surface area (mm^2^) stained for neutrophils (NEU), macrophages (MAC), mast cells (MAST), and eosinophils (EOS). (B) Number of NETs, METs, MCETs, and EETs per surface area (mm^2^). (C) Proportion of cells (NEU, MAC, MAST, and EOS) that undergo etosis (%). All types of ETs were present with various amounts. NETs were the most prominent traps, especially in fresh and lytic thrombi (*p < 0.05 to organised thrombi, *n* = 48).

Next, we investigated the presence and extent of different types of ETs in relation to the age of the coronary thrombus in thrombus aspirates. In line with the findings on autopsy specimens, all types of ETs could be detected. Cell‐specific differences in the source of traps were noticed when thrombus specimens of different ages were compared (see Figure 3 and supplementary material, Figure S3 for representative examples of NETs, METs, MCETs, and EETs in coronary thrombi). Neutrophils were the major source of extracellular traps (Figure 4) in fresh (151 per mm^2^) and lytic thrombi (136 per mm^2^), followed by METs in fresh (45 per mm^2^) and in lytic thrombi (99 per mm^2^). In organised thrombi, on the other hand, METs exceeded NETs in number (30 per mm^2^ versus 20 per mm^2^, Figure 4). The numbers of mast cell‐ and eosinophil‐derived traps were much lower, with most MCETs present in organised thrombi (6 per mm^2^, Figure 4) and EETs in lytic thrombi (7 per mm^2^, Figure 4).

## Discussion

In this study, we showed that not only neutrophils but also other types of leucocytes form extracellular traps (ETs) in atherosclerotic plaques and to a much larger extent in the coronary thrombus of MI patients. NETs and METs are the two most prominent contributors to etosis in coronary atherothrombosis, although low numbers of mast cell‐ and eosinophil‐derived traps (MCETs and EETs) can also be encountered in the lesions. When the age of a thrombus was taken into consideration, NETs appeared to dominate in the early (fresh) stages, whereas METs are significantly more numerous in the late (organising) stages of thrombus formation (see Figure 4 and supplementary material, Figure S3G–L). These findings imply that once etosis is considered as a process involved in the progression and maturation of coronary thrombus after its onset, different types of leucocytes participate in a time‐dependent manner.

### Macrophage extracellular traps (METs) in atherothrombosis

Recent studies have shown that macrophages also are capable of releasing extracellular DNA in response to micro‐organisms and inflammatory mediators 13, 34. *In vitro*, METs were visualised with the citrullinated histone H4 in IFN‐γ‐induced human monocyte‐derived macrophages upon *Mycobacterium tuberculosis* infection 15 and with citrullinated histone H3 in human peripheral monocytes infected with *Candida albicans* 35 *. In vivo*, a histological study on kidney biopsies from patients with ANCA‐associated vasculitis reported the presence of METs (identified as MPO^+^CD68^+^elastase^−^ macrophages co‐expressing CitH3) in glomerular MPO‐containing macrophages. In all those studies, similar antibodies were applied to those we used in the present sudy, to visualise histones as the key elements in the structure of extracellular traps 36.

In intact plaques, we observed significantly higher numbers of METs compared with the other types of ETs; they were mostly located around and inside the lipid core, which also contained immunostainable remnants of dead macrophages. Macrophage cell death, generally attributed to necrosis and apoptosis, is considered an important contributor to the growth of lipid cores of atherosclerotic plaques. Death of (foamy) macrophages leads to extracellular spill of lipids and, as a consequence, volume expansion of the lipid core (also termed the ‘graveyard of macrophages’) 37. Our study reveals that also ‘metosis’, as shown by the presence of METs, contributes to macrophage cell death and, as can be anticipated, to the expansion of the lipid‐rich atheroma of plaques which typifies the vulnerable type of lesions.

Still, in thrombosed coronary atherosclerotic plaques (be they ruptured or eroded), the numbers of METs were significantly increased compared with intact plaques, not only in the plaque or overlying thrombus but also in the periadventitial tissues surrounding these complicated plaques. As yet, it remains unknown whether METs contribute to the onset of thrombus formation or otherwise aggravate the process of progression of a thrombus, for example by forming a scaffold for fibrin network or stimulating other pro‐thrombotic and pro‐coagulant mediators. The significant number of METs that we found in the perivascular fat tissue around thrombosed plaques follows the pattern that we reported also for NETs previously 6. We interpreted the abundant presence of NETs in the adventitia and perivascular fat tissue as a sequential effect of acute plaque complications in which the periadvential inflammation is triggered by neutrophil activation and cell death. It could be that the same process applies for the formation of METs at this location. However, at present, we cannot say whether the formation of METs can be considered beneficial or detrimental in atherothrombosis, as macrophage functions are considerably dynamic according to their micro‐environment, differentiation, and polarisation states 23, 38.

### Mast cell and eosinophil extracellular traps (MCETs and EETs) in atherothrombosis

Several *in vitro* and *in vivo* studies have described the presence of MCETs and EETs mainly in infectious, allergic, and autoimmune diseases. *In vitro*, MCETs were first recognised on human mast cell‐1 (HMC‐1) infected with several bacteria 18, while EETs were noticed in human purified eosinophils primed with IL‐5 and IFN‐γ under the influence of lipopolysaccharide (LPS), complement (C5a) or eotaxin 39. *In vivo*, MCETs were identified in psoriatic lesions and in skin explant cultures treated with IL‐23 and/or IL‐1β by using immunofluorescence with tryptase and DAPI 17, while EETs were detected in the intestines of patients with Crohn's disease 20, in skin diseases 19, and in bronchial biopsies of asthmatic patients 40 with the use of DNA and EMBP immunostaining. Overall, the use of cell‐specific antibodies in those studies – tryptase for mast cells and EMBP for eosinophils – was also implemented in our study.

In thrombosed coronary atherosclerotic plaques (be they ruptured or eroded), we found only a relatively small number of MCETs and EETs, in line with the usually small number of mast cells and eosinophils, respectively, that have been reported in plaques, including the lesions of MI patients 41, 42. Still, despite their low number, mast cells attract interest because of the role that they play in the process of plaque destabilisation via their release of pro‐inflammatory cytokines and vasoactive mediators (histamine, chymase, and tryptase). It could be that the formation of traps by mast cells facilitated these functions 11, 13. Although to a lesser extent compared with thrombosed plaques, most mast cells have been reported in the adventitia of intact plaques around microvessels 43, 44, which is also the site where we observed most MCETs. Therefore, it is conceivable that their traps stimulate pro‐inflammatory functions in the periadventitial fat 45, 46, 47.

Furthermore, investigations into the underlying mechanisms of etosis other than netosis are currently less numerous 13, 34. The formation of ETs by neutrophils, macrophages, mast cells, and eosinophils may share some resemblances as well as differences. For example, the generation of reactive oxygen species (ROS) 13 and the involvement of enzymes (MPO, elastase or tryptase) in ET generation 18, 34 will likely be the same in neutrophils, macrophages, and mast cells. On the other hand, cytokines and chemokines involved in the generation of traps can be similar or different for the various types of leucocytes: for example, IFN‐γ and C5a in NETs and EETs; IL‐8 in NETs; and IL‐23 and IL‐1β in MCETs 13.

In conclusion, not only neutrophils but also macrophages, and to a lesser extent also mast cells and eosinophils, generate ETs after the onset of coronary plaque complications. In turn, the formation of ETs spans all stages of coronary thrombosis evolution. Different types of leucocytes and their ETs participate in orchestrating thrombus organisation and maturation towards stability in a time‐dependent manner. More knowledge about the role of ETs during atherothrombotic disease is important, since it may provide new strategies in the treatment of cardiovascular disease.

## Author contributions statement

KRP, OJB, and ACW designed the study. KRP and XL prepared and assessed the specimens. KRP and CM performed experiments. KRP, CM, and DP analysed the data. KRP, DP, and OJB contributed to manuscript design. KRP wrote the manuscript, while DP, OJB, RDW, and ACW critically revised the manuscript. OJB and ACW supervised this study. All authors had final approval of the submitted versions.

**SUPPLEMENTARY MATERIAL ONLINE**

**Supplementary figure legends**

**Figure S1.** Triple immunohistochemical staining to visualise NETs and METs simultaneously in a coronary thrombus

**Figure S2.** Control slides for immunohistochemical staining

**Figure S3.** Extracellular traps in different types of thrombus specimen

## Supporting information


**Supplementary figure legends**

**Figure S1.** Triple immunohistochemical staining to visualise NETs and METs simultaneously in a coronary thrombus. These figures show the same section stained sequentially with anti‐MPO (A), anti‐CD68 (B) and anti‐CitH3 (C) antibodies, with an elution step between staining rounds. (D) false‐colour image showing NETs as the colocalization of MPO^+^CD68^−^CitH3^+^ (in yellow) and METs as the colocalization of CD68^+^MPO^−^CitH3^+^ (in cyan). Scale bar in (A): 5 μm

**Figure S2.** Control slides for immunohistochemical staining. Representative examples of positive and negative control slides for CitH3 immunostaining, performed after an elution step to remove the previous dye and immune‐complexes, in two parallel sections of a coronary thrombus specimen. (A) Section stained with anti‐CitH3 antibody as positive control, showing positive cells in dark red. (B) Section stained with the omission of anti‐CitH3 antibody (substituted with antibody diluent only) as negative control, showing negative staining. Scale bar in (A): 50 μm

**Figure S3**. Extracellular traps in different types of thrombus specimen. Representative examples of neutrophil, macrophage, mast cell, and eosinophil extracellular traps (NETs [A‐F], METs [G‐L], MCETs [M‐R] and EETs [S‐X]) in all stages of thrombus evolution: fresh, lytic and organized. Boxed areas in H&E stains (A, C, E, G, I, K, M, O, Q, S, U, W) show the regions of interest for higher magnification of false‐colour images to show the co‐localization of cell‐specific markers (in red) with CitH3^+^ (in green). Colocalization appears in yellow in all false‐colour images. (B, D, F) NETs as MPO^+^CitH3^+^; (H, J, K) METs as CD68^+^CitH3^+^; (N, P, R) MCETs as tryptase^+^CitH3^+^; (T, V, X): EETs as EMBP^+^CitH3^+^. Scale bar in H&E overview (A): 100 μm and in high power detail (B): 25 μm
