# Supplementary figures and images for: Extracellular traps derived from macrophages, mast cells, eosinophils and neutrophils are generated in a time‐dependent manner during atherothrombosis
Source: J Pathol. 2019 Jan 25;247(4):505–12. doi: 10.1002/path.5212 (PMC6590313; doi:10.1002/path.5212)

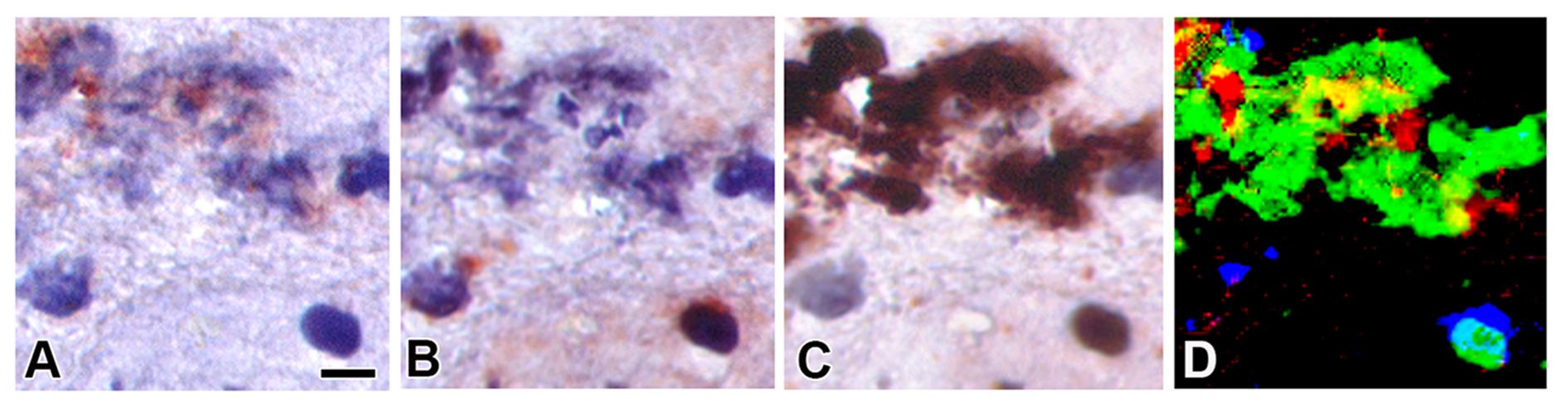

Supplement: Supplementary file 2 — Figure S1. Triple immunohistochemical staining to visualise NETs and METs simultaneously in a coronary thrombus. These figures show the same section stained sequentially with anti‐MPO (A), anti‐CD68 (B) and anti‐CitH3 (C) antibodies, with an elution step between staining rounds. (D) false‐colour image showing NETs as the colocalization of MPO+CD68−CitH3+ (in yellow) and METs as the colocalization of CD68+MPO−CitH3+ (in cyan). Scale bar in (A): 5 μm [file PATH-247-505-s003.tif]

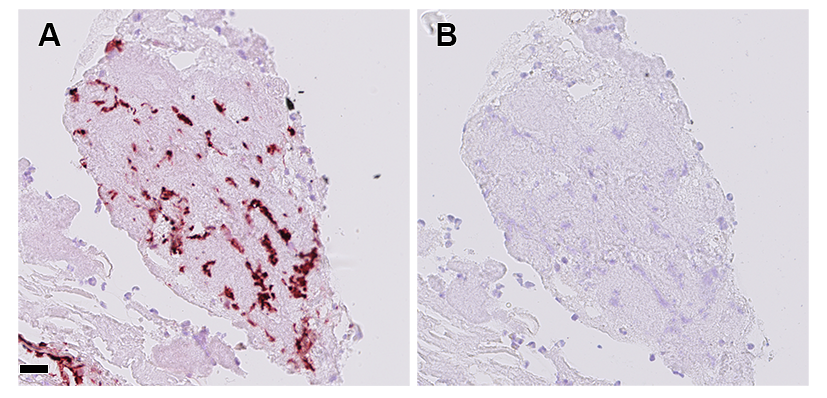

Supplement: Supplementary file 3 — Figure S2. Control slides for immunohistochemical staining. Representative examples of positive and negative control slides for CitH3 immunostaining, performed after an elution step to remove the previous dye and immune‐complexes, in two parallel sections of a coronary thrombus specimen. (A) Section stained with anti‐CitH3 antibody as positive control, showing positive cells in dark red. (B) Section stained with the omission of anti‐CitH3 antibody (substituted with antibody diluent only) as negative control, showing negative staining. Scale bar in (A): 50 μm [file PATH-247-505-s002.tif]

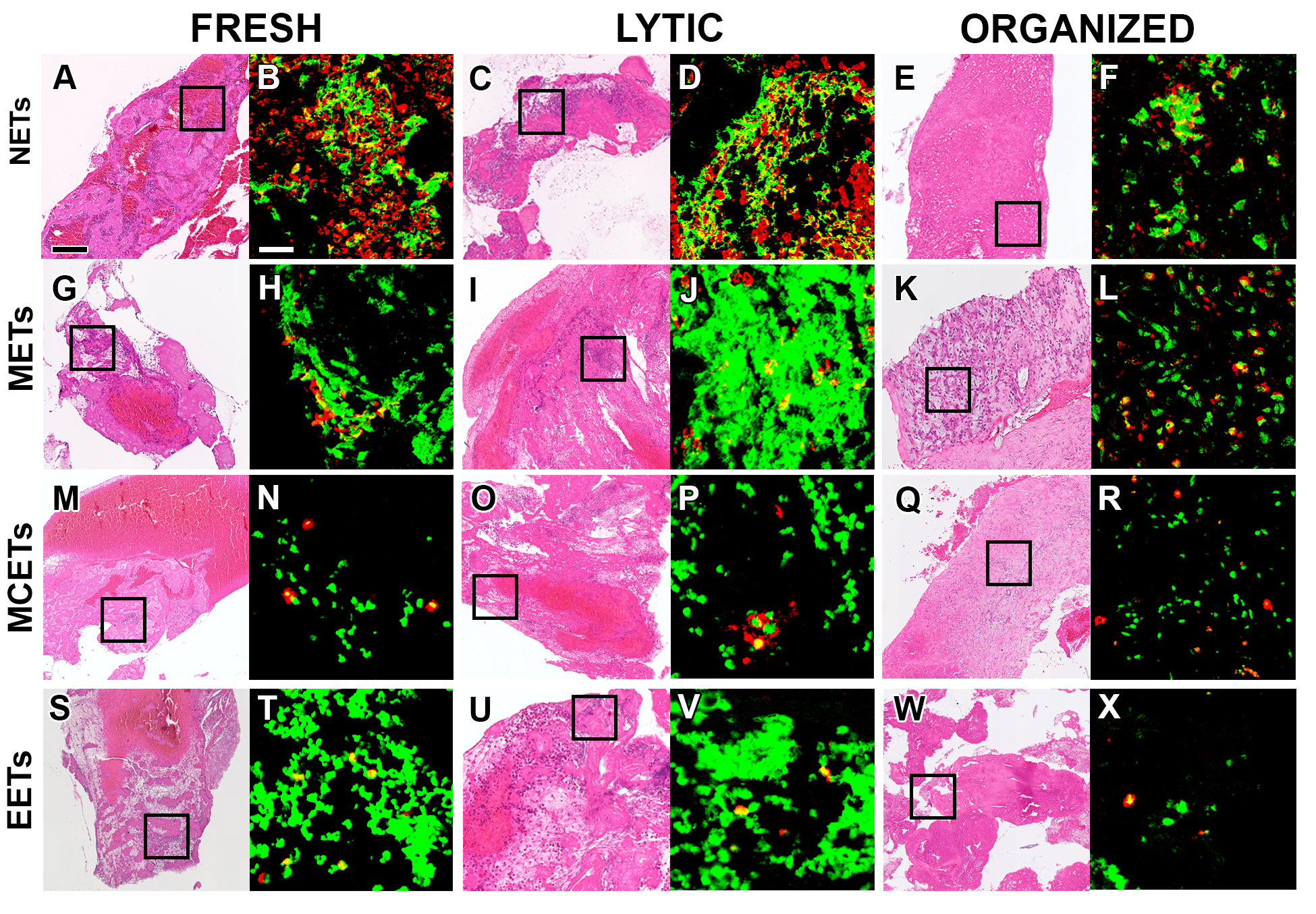

Supplement: Supplementary file 4 — Figure S3. Extracellular traps in different types of thrombus specimen. Representative examples of neutrophil, macrophage, mast cell, and eosinophil extracellular traps (NETs [A‐F], METs [G‐L], MCETs [M‐R] and EETs [S‐X]) in all stages of thrombus evolution: fresh, lytic and organized. Boxed areas in H&E stains (A, C, E, G, I, K, M, O, Q, S, U, W) show the regions of interest for higher magnification of false‐colour images to show the co‐localization of cell‐specific markers (in red) with CitH3+ (in green). Colocalization appears in yellow in all false‐colour images. (B, D, F) NETs as MPO+CitH3+; (H, J, K) METs as CD68+CitH3+; (N, P, R) MCETs as tryptase+CitH3+; (T, V, X): EETs as EMBP+CitH3+. Scale bar in H&E overview (A): 100 μm and in high power detail (B): 25 μm [file PATH-247-505-s001.tif]
